# Itch Processing in the Skin

**DOI:** 10.3389/fmed.2019.00167

**Published:** 2019-07-19

**Authors:** Martin Schmelz

**Affiliations:** Department Experimental Pain Research, CBTM, Medical Faculty Mannheim, Heidelberg University, Mannheim, Germany

**Keywords:** neuropathic itch, sensitization, pattern theory, itch pathway, degeneration, inflammation

## Abstract

Itching can result from activity of specialized primary afferent neurons (“pruriceptors”) that have been shown to express certain molecular markers such as B-type natriuretic peptide and several members of the Mrgpr-family in rodents. On the other hand, neurons involved in pain processing (“nociceptors”) can also provoke itching when the activation site is restricted to an isolated tiny spot within the epidermis. Individuals classified as having sensitive skin report increased itching and pain sensations upon weak external stimuli that are not painful or itchy in the control group. Numerous possible factors could contribute to sensitive skin along the pathway of transduction of the external stimuli into peripheral neuronal signals, followed by neuronal processing, finally resulting in the perception: (a) reduced local protective factors leading to impaired skin barrier function, (b) increased production of excitatory skin mediators, (c) sensitized peripheral neurons, (d) facilitated spinal and central processing, and (e) reduced descending inhibition from the central nervous system. For all of those pathophysiological mechanisms there are clinical examples such as atopic dermatitis (a,b,c), neuropathic itching (c,e), and restless leg syndrome (d,e). However, none of these factors have been directly linked to the occurrence of sensitive skin. Moreover, individuals reporting sensitive skin are heterogeneous and a subpopulation with defined pathophysiology has not yet been identified. Given that the condition is reported in about 50% of women, and thereby includes many healthy individuals, it appears problematic to assign a definitive pathophysiological mechanism to it.

## Itch Processing and Sensitive Skin

Sensitive skin has recently been defined “by the occurrence of unpleasant sensations (stinging, burning, pain, pruritus, and tingling sensations) in response to stimuli that normally should not provoke such sensations. These unpleasant sensations cannot be explained by lesions attributable to any skin disease. The skin can appear normal or be accompanied by erythema” (1). Concerning the occurrence of itching in sensitive skin, possible contributing factors could interact all the way between barrier function, peripheral neuronal activation, central processing, and descending inhibition. In this manuscript the neurophysiology of itch processing will be summarized and possible interactions in sensitive skin will be discussed.

## Molecular Markers for Itch Processing Neurons

Specific pathways have been identified in rodents that are involved in encoding non-histaminergic itching. These include functional markers for primary pruriceptive afferent neurons in rodents (MrgprA1, MrgprC11, MrgprD) and human (MrgprX1) (2), peripheral mediators that are primarily linked to itching rather than pain behavior (interleukin [IL] 13, IL-31, autotaxin, lysophosphatidic acid [LPA], thymic stromal lymphopoietin [TSLP], cathepsin S) (3) and central transmitters and pathways for itch processing (B-type natriuretic peptide [BNP], gastrin releasing peptide [GRP]) (4, 5). Spinal neurons containing gastrin releasing peptide have been found critical for itching, but not for pain (6) suggesting specificity, even though they are also activated by nociceptive stimuli (7). However, it is important to avoid over-simplification as many of the itch-related mediators, receptors or markers are also found in nociceptive pathways. This is obvious for the neurokinin 1 receptor (NK1R) (8), sodium channel subtypes, such as NaV1.7 (9, 10) and LPA (11). Moreover, MrgprD positive neurons originally described as nociceptors (12) have recently been implicated in neuropathic pain (13) and neurons carrying the TSLP receptor were described as mediating “itch and/or pain” (14).

### Specificity

About 20 years ago, mechanoinsensitive (“silent”) histamine-sensitive C-nociceptors in human (15) and spinothalamic projection neurons in a cat (16) were identified as part of a specific pruritic pathway. More recently, molecular markers of non-histaminergic itch-specific neurons were identified in rodents, such as B-type natriuretic peptide (BNP) (17, 18) and members of the mas-related G-protein receptor family (mrgprA3, C11) (19–21) in primary afferent neurons, but also the gastrin releasing peptide (GRP) (5, 7, 22) in dorsal horn neurons. Non-histaminergic itch signaling has received major interest when mas-related G-protein coupled receptors (Mrgprs) were identified on presumably itch-related neurons in mice, i.e., MrgprA3 (23), D (24), and C11 (25). Also, BAM8-22, an activator of MrgrpC11 induces itching in the human skin (26). Similarly, an intracutaneous injection of beta-alanine, an activator of MrgprD, provokes mainly itching, but also pain in humans (24, 27). Chloroquine has often been used in mice to elicit itch-behavior via the activation of MrgprA3 (23, 28, 29). Thus, the plethora of new information on pathways and mediators for itching in rodents as described above might imply that the “labeled line” theory for itching has finally been verified.

**Figure 1:**
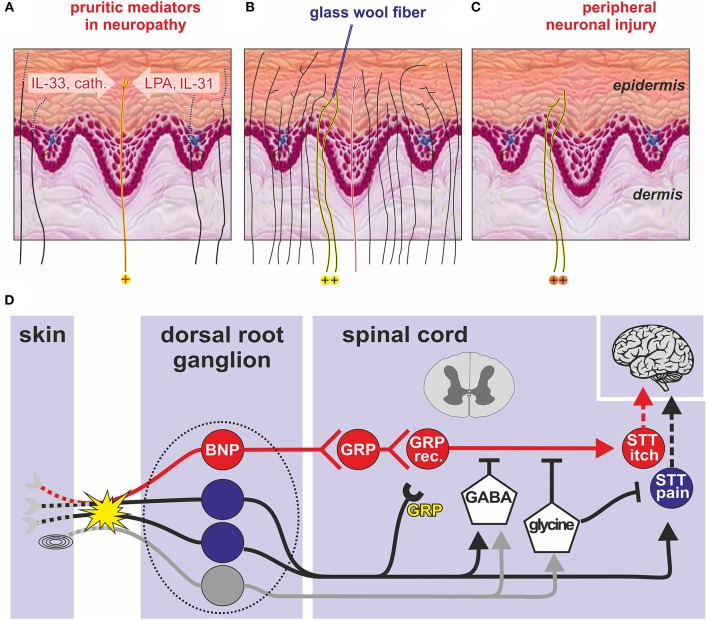
Transduction of an itch in the skin **(A-C)**. **(A)** Degenerating peripheral nerve fibers (dotted lines) due to peripheral sensorineural injury may release inflammatory mediators such as LPA, IL-31, and IL33 (30) that activate itch-specific pruriceptors (red line, labeled yellow with “+” at bottom). The pruriceptors cause itching via activation of itch-specific pathways (red “labeled line”). **(B)** In healthy skin, itching can be caused when punctate stimuli (e.g., a glass wool fiber) activate only a few adjacent nociceptive fibers within the epidermis (labeled yellow with “+” at bottom) whereas directly adjacent fibers, including specific pruriceptors (red) remain silent. If instead activated en masse (e.g., by trauma) their combined activation will cause pain. **(C)** The same localized activation can be mimicked after peripheral neuronal injury when spontaneous action potentials from the few remaining abnormal epidermal nociceptors (labeled yellow with “+” at bottom) reproduce the discharge profile of non-lesioned skin (“spatial contrast mechanism” of itching). **(D)** Spinal processing of itching based on animal data: Skin and mucosal BNP primary sensory neurons (red) with cell bodies in the dorsal root ganglion (dotted line) stimulate GRP-releasing interneurons in the dorsal horn of the spinal cord that stimulate GRP-receptive interneurons (GRP rec.) and finally projection neurons (STT itch) that send itching signals to the brain via the contralateral STT. Pain neurons (blue) and touch neurons (gray) can inhibit ascending itch signals via GABAergic interneurons, whereas glycinergic interneurons inhibit both itching and pain processing. Peripheral nerve injury (yellow explosion) can induce GRP *de-novo* synthesis that might facilitate spinal itch processing (yellow “GRP”). BNP, B-type natriuretic peptide; cath., cathepsin S; DRG, dorsal root ganglion; GABA, gamma aminobutyric acid; GRP, gastrin related peptide; IL-31, interleukin 31; IL-33, interleukin33; LPA, lysophosphatidic acid; SST, spinothalamic tract, Pacinian corpuscles (symbol before the yellow explosion) modified from Steinhoff et al. (33).

If itching in sensitive skin was based on the activation of specific “pruriceptors” the peripheral pruritic mediators released in sensitive skin and the neuronal pathway needs to be identified. One could hypothesize that like in neuronal degeneration, inflammatory mediators (30), such as interleukin-31 (IL-31), IL-33 (30), lysophosphatidic acid (LPA), and cathepsin S are released (31, 32) from degenerating axons, which could selectively activate pruriceptors as schematically shown in Figure 1A. Although such a scenario appears plausible, LPA (34), cathepsin S (31), and IL-33 (35) have also been linked to chronic pain conditions, and thus, might not be optimally suited as itch-specific mediators. Only IL-31 would remain as mediator, mainly associated to itching rather than pain, which would be in line with human data on antipruritic effects of IL-31 receptor antibodies (36), but also the co-expression of the IL-31 receptor and natriuretic peptide B in a subpopulation of dorsal root ganglion neurons (37). Thus, for inflammatory itching with high levels of TH-derived IL-31, such as atopic dermatitis, human data also suggests a simple itch pathway in which peripheral pruritogens activate a specific pruriceptive subpopulation of neurons.

## Spatial Contrast Type of Itch

Electrophysiological data from rodents and monkeys did not support a “labeled line” for itching (38–40) as no specific subpopulation of itch neurons was found. The results rather support the pattern theory of itching according to which nociceptors can signal an itch or pain based on the combination of activated fibers, resulting in population coding (41). Moreover, very focal activation of nociceptors in the skin can explain itching without a “labeled line”: noxious stimulation that is directed only to a few sensory endings within the epidermis elicits itching in human skin (42), even when the stimulus is the algogen capsaicin. It has been suggested that local activation of only few epidermal nociceptors can cause itching by a “mismatch signal” (43) or “spatial contrast” (44), provided by few activated and many non-activated nociceptive endings innervating the same skin site, for example by a minute glass wool fiber (Figure 1B). Such a discharge pattern indicates that a noxious event is minute and localized within the epidermis.

In peripheral neuropathy the skin is partially denervated leaving some isolated sensory endings within the epidermis (Figure 1C). Clinically, we used to associate such depletion of skin innervation to reduced sensory function. However, if there is some local inflammation or spontaneous activity of these isolated remaining nerve branches, the resulting discharge pattern would equal the one just described as “spatial contrast.” Thus, a combination of ongoing or evoked activity from sparsely surviving or newly regenerating nerve branches could generate neuropathic itching via the “spatial contrast” mechanism.

It is of clinical interest that the same spatial arrangement of isolated epidermal sensory nerve fibers is generated by neurons reinnervating scar tissue, for example after burns (45). The combination of spatial arrangement and spontaneous activity of regenerating sprouts might underlie the development of an itch in this condition. Of note, scratching itself can also lead to the reduction of epidermal nerve fiber density (46, 47); however, it remains unclear to which extent scratch-induced axotomy might exaggerate chronic inflammatory itching conditions.

It is remarkable that it took only about 10 years to identify an itch-specific spinal pathway in mice: B-type natriuretic peptide (BNP) skin afferents in the periphery, synapse onto BNP receptor positive neurons in the superficial dorsal horn that contain gastrin releasing peptide (GRP). The third neuron expresses GRP-receptors and excites pruriceptive neurons that ascend the spinothalamic tract (48) (Figure 1D). Itchiness is one of the most common side-effects of administering drugs targeting μ-opioid receptors, and this seems to be related to cross-activation of spinal GRP receptors (49).

In mice, peripheral nerve injuries incite broad *de-novo* GRP expression in DRG neurons (50). This response might contribute to neuropathic itching as nociceptors that typically inhibit itching may undergo a phenotypic switch by this *de-novo* expression. When they become spontaneously active, they could release their GRP in the dorsal horn and contribute to neuropathic itching via volume transmission (Figure 1D, yellow “GRP”). Thus, in addition to the spatial contrast mechanism described above, peripheral nerve injury could also provoke neuropathic itching via *de-novo* expression of GRP in primary afferent nociceptors.

## Combination of Itch Theories

**Figure 2:**
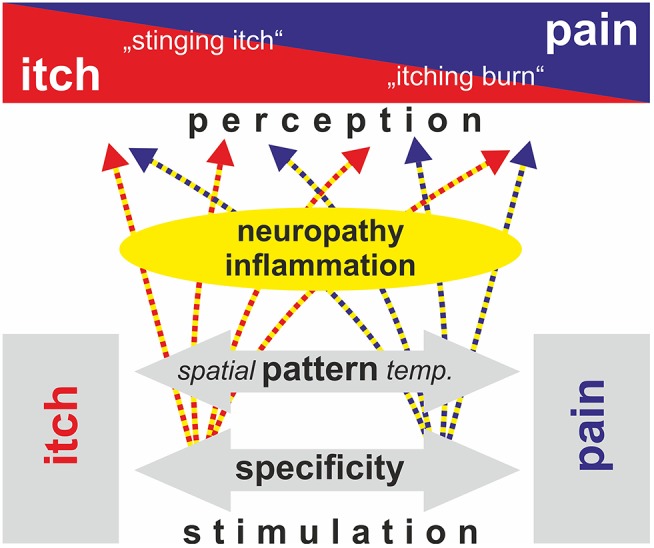
Schematic view of mechanisms that have been proposed to explain the generation of an itch (specificity, pattern, intensity; black and white boxes). Neuropathy and inflammatory processes can modulate primary afferent discharge and spinal processing such that spatial and temporal pattern are changed (gray arrow). Thereby discharge that would genuinely be processed as distinct itching may be perceived as partly painful (“stinging itch”) and vice versa (“itching burn”). Rather than strictly following only one of the above theories, the generation of neuropathic itching in patients thereby may be based on a combination of spatial/temporal pattern and specificity. Modified from Steinhoff et al. (54).

Basic itching mechanisms are generally discussed in their “pure” form. However, as shown above, there is evidence that physiologic itch processing may combine elements from different basic theories traditionally regarded as mutually exclusive. Under experimental conditions, more of such combinations appear consistent with experimental data: activation of subpopulations of C-afferents, such as MrgprA3 positive nociceptors (6, 51), might generate itching not only via the assumed specificity, but will also activate a subset of nociceptors innervating a given skin site. Such a combination of active and non-active nociceptors from the same skin site will thereby mimic a spatial contrast pattern. Another example would be a high number of spontaneously active nociceptors that signal pain; upon reduction of this number, for example by analgesic therapy, the chances increase to create a spatial contrast pattern by the still active nociceptors. This would represent a combination of a spatial contrast theory and the old intensity theory. Interestingly, clinical observations indeed support such a development: in patients with postherpetic neuralgia, resolving pain may be combined with an increase in itching (52, 53). Thus, based on defined experimental models, we have successfully developed basic theories that can explain differentiation between itching and pain based on specificity in a “labeled line” or the discharge pattern in its temporal or spatial expression (Figure 2, lower part). These approaches provide us with powerful tools when trying to explain a clinical neuropathic itch. However, rather than assuming that in pathological conditions there is a mutually exclusive explanation for itching purely based on one theory of itching, we might rather adapt our conceptual framework and include mechanisms that contribute elements from several theories (Figure 2, upper part).

## Potential Links Between Sensitive Skin and Facilitated Itching

Even though there is a correlation with sensitive skin and a dry skin type (55) it is not included in the definition of sensitive skin (1). Dry skin models of itching (56) can therefore not be directly linked to sensitive skin. Thus, hypotheses related to neurons have to be discussed: sensitized peripheral neurons alone or in combination with facilitated spinal processing could underlie sensitive skin. Alternatively, central processing alone or in combination with reduced descending inhibition from the central nervous system could be key mechanisms.

## Peripheral Neuropathy and Sensitive Skin

Peripheral neuropathy is a common cause of neuropathic pain and itching (33, 54) with postherpetic neuralgia and diabetes being abundant examples. The diagnosis of small fiber neuropathy can be based on reduced epidermal innervation density (57) and functional impairment as measured by quantitative sensory testing (QST) (58). There is ample evidence for a clear correlation between altered sensory thresholds and epidermal nerve fiber density: very high correlations (up to 0.75) were found between QST parameters, intraepidermal nerve fiber density and sensory scores (59) and in general, QST has proven to be a sensitive and highly useful tool for early detection of small fiber impairment (without pain) in diabetes (60). On the one hand the link between sensory thresholds and itching or pain is more problematic. The severity of small fiber neuropathy does not predict painfulness or itching in the patients (61) as not a single item from the QST battery was helpful in differentiating neuropathic patients with severe pain from those without pain (62). Even without this clear link it is remarkable that individuals with sensitive skin had lower innervation densities as compared to control subjects (17 vs. 15 fibers per mm) (63), but innervation density was still in the normal range; for small fiber neuropathy one would expect more pronounced reductions below six fibers per mm (64). Functional tests revealed sensitized heat pain thresholds in sensitive skin individuals (65) and links between sensitive skin and small fiber neuropathy were proposed (63, 66–68). However, based on the negative correlation between epidermal innervation density and heat pain thresholds small fiber neuropathy would be expected to increase rather than decrease heat pain thresholds (61). Thus, based on functional and structural results, there is no evidence for small fiber neuropathy in individuals with sensitive skin.

### Central Processing and Descending Inhibition

It has been noted that patients with sensitive skin also report a sensitive cornea (69) and irritable bowel syndrome (70), that is also linked to interstitial cystitis (71), and fibromyalgia (72). Increased pain sensitivity is observed in all these conditions and central sensitization has generally been assumed as a common mechanism (73, 74). In this respect it is interesting that definitive small fiber neuropathy with pathologically reduced intraepidermal nerve fiber density has been reported in patients with fibromyalgia (75, 76) possibly indicating a special subpopulation with peripheral neuropathy. In chronic pain the importance of catastrophizing has been established in recent years (77) with catastrophizing also correlating to ratings of induced pain (78). Based on the overlap between sensitive skin and the above-mentioned chronic pain conditions, one might expect that there is also some overlap in terms of higher scores for catastrophizing. Recent data in mice even provide direct evidence for an overlap between itching and anxiety-like behavior when histamine-responsive central neurons in the amygdala were stimulated optogenetically (79). Unfortunately, there is currently no data available on catastrophizing or anxiety in individuals reporting sensitive skin. Moreover, when separating peripheral vs. central neuronal processing we over-simplified and did not take into consideration interactions that exist between central stress responses and cutaneous neuro-immune interactions (80).

Increased evoked mechanical pain ratings were also found in restless leg syndrome (81), a condition in which reduced descending inhibition has been assumed (82). In primary restless leg syndrome typical mechanical pinprick stimuli are felt more intensely, but there is no evidence for small fiber impairment such as heat hypoalgesia (83, 84). Interestingly, centrally acting analgesics such as gabapentin have been successfully used for treatment (85). In summary, centrally mediated hypersensitivity has been established as a contributing factor in a number of chronic pain conditions. Thus, reduced descending inhibition or broadly increased central processing can lead to hypersensitivity to noxious stimulation. However, it is unclear to which degree such a phenomenon contributes to sensitive skin.

## Perspectives

Given the fast progress in our understanding of itch mediators, pathways and processing we might feel to be close to link those mechanisms to sensations reported in sensitive skin. However, to date there is no clear evidence for any specific changes in the itching pathway that could explain itching in sensitive skin. The link between sensitive skin and irritable bowel syndrome might suggest that sensitized central processing or reduced descending inhibition similarly contributes to both conditions. It is important to note that research on sensitive skin has mainly focused on peripheral mechanisms whereas it is established that central processing is highly important for chronic pain or itch conditions. Moreover, in the absence of objective pathologic findings, such a common complaint should not be regarded as abnormal or diseased, but as within the normal range. Given that a high percentage (about 50%) of the population reporting sensitive skin, we would expect a high degree of heterogeneity and therefore studies should include parameters affecting central processing of sensory information such as catastrophizing.

## Author Contributions

The author confirms being the sole contributor of this work and has approved it for publication.

### Conflict of Interest Statement

The author declares that the research was conducted in the absence of any commercial or financial relationships that could be construed as a potential conflict of interest.
